# Therapeutics, Etc.

**Published:** 1890-11

**Authors:** 

**Affiliations:** Austin


### Items of Interest.

[CULLED BY DR. CHURCH, AUSTIN.]

**Pilocarpine for Chronic Bronchitis.**—In the treatment of ordinary cases of bronchial catarrh, accompanied by cough, arrested secretions derangement of digestion, etc., etc., pilocarpine in small doses at short intervals, says the Medical Summary is one of the most effective remedies that we have at the present time, and when in the form of tablet triturate can be given to children, adults, and all persons without regard to the stomach.

The dose for adults should not be more than one-fiftieth of a grain every hour or two, and the preparation should be allowed to dissolve in the mouth. Not alone the cough and bronchial secretions are improved, but the activity of the salivary glands is increased, and this increased secretion entering the stomach the functions of that organ are improved, and in the course of a day or two the condition of the patient is changed in a remarkable manner.—[N. Y. Medical Times.

**The Influence of Alcohol on a Healthy Stomach.**—In order to elucidate the effects of alcohol on the gastric digestion in healthy subjects, Dr. E. B. Blumanan, of St. Petersburg, has carried out a long course of elaborate experiments on five young men, aged from 22 to 24. Alcohol was used in the shape of a 25 and 50 (vol.) per cent. solution, of which 100 cub. cent. were taken ten or twenty minutes before the patient's dinner (consisting of soup, cutlet and bread).

The following are the main results arrived at by the author.

1. During the first three hours after the ingestion, the gastric digestion is markedly retarded, which is dependent upon diminished digestive power of the gastric juice, in other words, upon a decrease in the proportion of hydrochloric acid present therein.

2. The diminution is especially pronounced in persons non-habituated to the use of alcohol.

3.. Stronger solutions of alcohol act more energetically than weaker ones.

4. During the fourth, fifth and sixth hour after the meal digestion becomes considerably more active, the proportion of hydrochloric acid markedly rising.

5. Under the influence of alcohol the secretion of the gastric juice becomes more profuse and lasts longer than under normal conditions.

6. The motor and absorptive powers of the stomach, however, are markedly depressed, the decrease being directly proportionate to the strength of alcohol solutions ingested.

7. Alcohol distinctly retards the passage of food from the stomach to the duodenum.

8. On the whole, alcohol manifests decidedly unfavorable influence on the course of normal gastric digestion. Even when ingested in relatively small quantities, the substance tends to impair all gastric functions.

9. Hence a habitual use of alcohol by healthy people cannot possibly be approved of from a physiological standpoint. Dr. Blumenan, however, is far from repudiating alcohol in medicine.—[St. Louis Medical and Surgical Journal.

---

Aristol.—Dr. Wm. Waugh, in the Philadelphia *Times and Register* of September 20, giving his varied experience, and that of many others, in the use of this new addition to our remedial agents. Aristol, or dithymoldiodide, is a combination of iodine and thymol; proportion of iodine about 45 per cent. It is readily soluble in ether and oils, when rubbed in the latter, slightly soluble in alcohol. Heat and light should be avoided as they both decompose aristol. It is non-irritant, and is not absorbed into the body, as would seem from the fact that iodine has never been found in the urine after its tree use in wounds and ulcers. The general concensus of opinion of those who have used this new remedy, is that it has many claims over iodol, and being odorless, bids fair to supercede iodoform. Several cases are reported where iodoform caused irritation and was happily replaced by aristol. The pain and fetor of epithelioma of the vagina and uterus was removed, and it seemed the progress of the disease restored by applying tampons of cotton thickly dusted with aristol. five to ten per cent. aristol ointment gave good results in eczema, psoriasis, lupus, old ulcers, etc., etc.

---

**New York Pasteur Institute.**—Dr. Paxl Gibier, Director of the New York Pasteur Institute, New York, announces the results of the preventive inoculations against hydrophobia performed at this Institute since its opening (February 18, 1890,) as follows:

To date 610 persons, having been bitten by dogs or cats, came to be treated. These patients may be divided in two categories:

1st. For 480 of these persons it was demonstrated that the animals which attacked them were not mad. Consequently the patients were sent back after having had their wounds attended, during the proper length of time, when it was necessary. *400 patients of this series were consulted or treated gratis.*

2d. In 130 cases the antihydrophobic treatment was applied, hydrophobia having been demonstrated by veterinary examination of the animals which inflicted bites or by the inoculation in the laboratory, and in many cases by the death of some other persons or animals bitten by the same dogs. All these persons are, to-day, enjoyinging good health. *In 80 cases the patients received the treatment free of charge.*

The persons treated were: Sixty-four from New York, twelve from New Jersey, twelve from Massachusetts, eight from Connecticut, nine from Illinois, three from Missouri, three from North Carolina, three from Pennsylvania, two from New Hampshire, two from Georgia, two from Texas, and one each from Maryland, Maine, Kentucky, Ohio, Arizona, Iowa, Nebraska, Arkansas, Louisiana and Ontario, (Can.)

